# Longitudinal Trajectories of Health as Precursors of Average Levels and Temporal Patterns of Self-Continuity

**DOI:** 10.1093/geroni/igaf122.3071

**Published:** 2025-12-31

**Authors:** Yi Lu, Urmimala Ghose, Denis Gerstorf, Corinna Löckenhoff

**Affiliations:** Cornell University, Ithaca, New York, United States; Humboldt University in Berlin, Humboldt University in Berlin, Berlin, Germany; Humboldt University Berlin, Humboldt University Berlin, Berlin, Germany; Cornell University, Ithaca, New York, United States

## Abstract

Self-continuity, the sense of being the same person across the past, present, and future, is associated with physical and mental health and sensitive to prior life experiences. However, little is known about the association between self-continuity and longitudinal health trajectories across the adult life span. In this pre-registered study, we used data from 1,655 participants (aged 18-94, M = 52.73, 53% women) in the German Socio-economic Panel Innovation Sample. We examined the implications of longitudinal trajectories of mental and physical health for subsequent self-continuity—both with respect to average self-continuity and with respect to temporal patterns of self-continuity (i.e., relative self-continuity for past vs. future and close vs. distant time points). Self-continuity was assessed across three temporal distances (1/5/10 years) into the past and future. Health-related predictors captured both broad variations in general mental and physical health (i.e., life satisfaction and self-rated health) and more specific aspects (e.g., recent affective states, illness burden). Latent growth models were applied to examine the effects of longitudinal health trajectories on both average levels and temporal variations of past/future self-continuity. Multiple health factors, including illness burden, self-rated health, physician visits, life satisfaction, and affective states, significantly predicted both average levels of self-continuity and decrements in self-continuity for more distant time points. Interactions with age and gender were observed, and, consistent with prior research, self-rated health and negative affect had a stronger impact on past versus future self-continuity. Findings extend our understanding of longitudinal shifts in health as precursors of self-continuity across the life span.

